# Development and Validation of a Deep Learning Classifier Using Chest Radiographs to Predict Extubation Success in Patients Undergoing Invasive Mechanical Ventilation

**DOI:** 10.3390/bioengineering11060626

**Published:** 2024-06-19

**Authors:** Pranai Tandon, Kim-Anh-Nhi Nguyen, Masoud Edalati, Prathamesh Parchure, Ganesh Raut, David L. Reich, Robert Freeman, Matthew A. Levin, Prem Timsina, Charles A. Powell, Zahi A. Fayad, Arash Kia

**Affiliations:** 1 Department of Medicine Division of Pulmonary, Critical Care, and Sleep Medicine, Icahn School of Medicine at Mount Sinai, New York, NY 10029, USA; 2 Institute for Healthcare Delivery Science, Icahn School of Medicine at Mount Sinai, New York, NY 10029, USA; kim-anh-nhi.nguyen@mountsinai.org (K.-A.-N.N.); masoud.edalati@mountsinai.org (M.E.); prathamesh.parchure@mountsinai.org (P.P.); ganesh.raut@mountsinai.org (G.R.); robert.freeman@mountsinai.org (R.F.); prem.timsina@mssm.edu (P.T.); arash.kia@mssm.edu (A.K.); 3 Department of Anesthesiology, Perioperative and Pain Medicine, Icahn School of Medicine at Mount Sinai, New York, NY 10029, USAmatthew.levin@mssm.edu (M.A.L.); 4 Department of Genetics and Genomic Sciences, Icahn School of Medicine at Mount Sinai, New York, NY 10029, USA; 5 Windreich Department of Artificial Intelligence and Human Health, Icahn School of Medicine at Mount Sinai, New York, NY 10029, USA; 6 BioMedical Engineering and Imaging Institute, Icahn School of Medicine at Mount Sinai, New York, NY 10029, USA; 7 Department of Radiology, Icahn School of Medicine at Mount Sinai, New York, NY 10029, USA

**Keywords:** machine learning, artificial intelligence, deep learning, transfer learning, respiratory failure, mechanical ventilation, ventilator liberation, clinical decision support

## Abstract

The decision to extubate patients on invasive mechanical ventilation is critical; however, clinician performance in identifying patients to liberate from the ventilator is poor. Machine Learning-based predictors using tabular data have been developed; however, these fail to capture the wide spectrum of data available. Here, we develop and validate a deep learning-based model using routinely collected chest X-rays to predict the outcome of attempted extubation. We included 2288 serial patients admitted to the Medical ICU at an urban academic medical center, who underwent invasive mechanical ventilation, with at least one intubated CXR, and a documented extubation attempt. The last CXR before extubation for each patient was taken and split 79/21 for training/testing sets, then transfer learning with k-fold cross-validation was used on a pre-trained ResNet50 deep learning architecture. The top three models were ensembled to form a final classifier. The Grad-CAM technique was used to visualize image regions driving predictions. The model achieved an AUC of 0.66, AUPRC of 0.94, sensitivity of 0.62, and specificity of 0.60. The model performance was improved compared to the Rapid Shallow Breathing Index (AUC 0.61) and the only identified previous study in this domain (AUC 0.55), but significant room for improvement and experimentation remains.

## 1. Introduction

Endotracheal intubation and invasive mechanical ventilation (IMV) are lifesaving methods of support in patients with respiratory failure; however, IMV itself provides a significant risk of ventilator-related adverse events and must be discontinued as soon as possible [1]. Patient selection, timing of extubation, and liberation from IMV are challenging, and poor timing resulting in re-intubation increases ICU length of stay and mortality [2,3]. In general, clinician performance in this regard is poor, and a number of indices, called weaning predictors, to predict extubation outcomes have not resulted in a viable gold standard above clinical gestalt [4,5]. The increasing use of Machine Learning (ML)-based clinical decision support has resulted in a handful of ventilator liberation predictive engines using tabular data; however, these have proved difficult to operationalize and represent only a small amount of the breadth of clinical information bedside clinicians use in weaning assessment [6,7,8,9]. Deep learning has enabled ML-based decision support in images, and its use in the interpretation of chest X-ray (CXR) imaging is rising. Most work on ML in CXR imaging has focused on the diagnosis and identification of imaging abnormalities, with some models rising to radiologist-level accuracy [10]. This represents an opportunity for deep learning to extract information from CXR for higher-order decision support, such as prediction of extubation attempts and optimal patient selection for weaning. To our knowledge, only one small study has been conducted regarding the use of CXR to predict extubation outcomes, which was limited by poor predictive power compared to existing benchmarks [11]. Here, we develop and validate a model using deep learning and transfer learning to predict 72 h extubation success or failure in medically critically ill patients on IMV.

## 2. Materials and Methods

### 2.1. Study Setting, Population, and Data Sources

**Figure 1:**
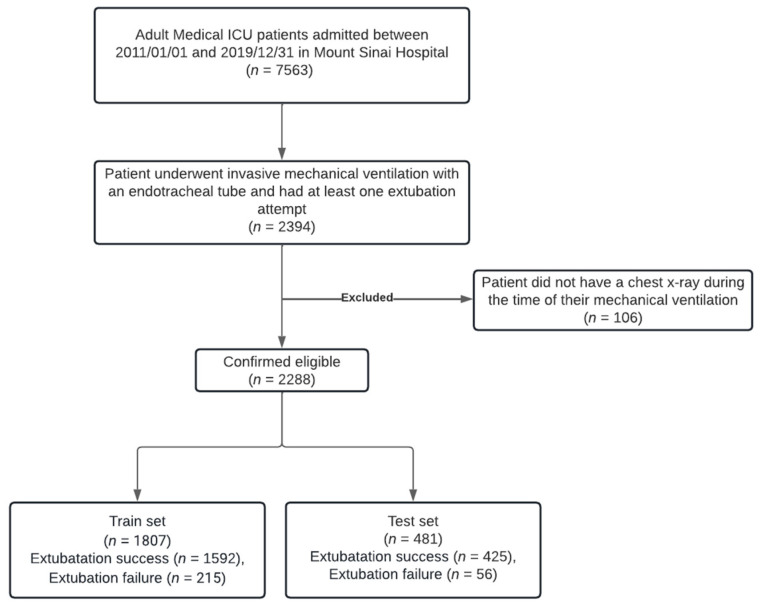
Patient flow and inclusion/exclusion criteria.

This study was undertaken at the Mount Sinai Hospital (MSH), an urban 1,134-bed tertiary-care teaching facility. We included adults (age ≥ 18 years) admitted to the Medical Intensive Care Unit from 1 January 2011 to 31 December 2019, who underwent mechanical ventilation via an endotracheal tube and had at least one extubation attempt. Patients lacking chest X-rays during ventilation or those palliatively extubated were excluded. Only a patient’s first extubation attempt per hospital visit was included in the dataset. All patients underwent regular assessments for ventilator liberation as per institutional protocol. This study identified extubation failures (re-intubation within 72 h) and successes (no re-intubation after 72 h). Figure 1 shows the flow chart for inclusions and exclusions for the cohort.

Data were collected from three EHR platforms—Epic (Epic Systems, Verona, WI, USA), Cerner (Cerner Corporation, North Kansas, MO, USA), and GE PACS. To assemble the chest radiograph dataset, we obtained raw DICOM (Digital Imaging and Communications in Medicine) files from the GE PACS platform. CXRs taken in both supine and upright positions were included. This study adhered to the Transparent Reporting of a multivariable prediction model for individual prognosis or diagnosis statement [12]. All methods were performed in accordance with relevant guidelines and regulations provided by the Institutional Review Board (IRB), which granted a waiver of informed consent (IRB-18-00573-MODCR001).

### 2.2. Image Pre-Processing

DICOM images were processed in an end-to-end automated fashion to prepare images for transfer learning and optimize model accuracy. Images were cropped to the smallest bounding box to remove irrelevant background noise. The images were then resized to 224 × 224 pixels and pixel intensities were rescaled to the (0, 255) SI range to fit the specifications of common pre-trained deep learning models. Histogram matching was used to standardize pixel intensities across scans and modalities. Given that the primary region of interest (ROI) in this study was the lungs, image segmentation was performed in an automated fashion using a pre-trained U-Net model architecture, LungVAE, trained on publicly available CXR datasets [13]. Centroid image alignment was performed to ensure critical lung structures remained centralized and resistant to rotation or other transformation.

### 2.3. Transfer Learning and Fine Tuning

We divided our cohort into two subsets: a training set (79% of the data) and a test set (21%), ensuring no patient overlap to validate our evaluation process effectively. Because of significant class imbalance (88% majority class, 12% minority class), we employed oversampling techniques on the training dataset, augmenting the minority class to balance the class representation using standard methodologies such as rotation (up to 15 degrees), horizontal flipping, translating, and random blurring.

ResNet50 was chosen over other CNN models like VGG, DenseNet, AlexNet, and GoogleNet due to its superior accuracy, reduced model complexity, and lower memory and computational demands [14]. Its use of residual connections helps in learning complex features efficiently, marked by its lower G-FLOP rates, making it ideal for our needs. The segmented CXRs were processed using a pre-trained ResNet50 model, modified for grayscale images and binary classification. Adjustments included a new convolutional layer with ReLu activation, dropout (0.4), and a sigmoid output function. Optimization was performed using the Adam optimizer, with initial settings of 18 epochs, a batch size of 25, and a learning rate of 0.001.

Using transfer learning and ten-fold cross-validation integrated with a grid-search algorithm refined the hyperparameters (learning rate, hidden units, dropout, batch size, epoch size) based on the AUROC [15]. Optimal settings were established as follows: 20 epochs, 0.2 dropout, batch size 16, learning rate 1 × 10^−3^, and 12 hidden units. Final model re-training and predictions were executed on the Pytorch framework (version 1.6.0) using GPU resources on Microsoft Azure’s cloud platform with 56 GB RAM.

### 2.4. Pixel Visualization

To interpret the image classifier’s results, we utilized the Grad-CAM technique, which produces class-specific heat-maps that highlight the influential areas in the CXRs for the model’s decision-making [16]. This method leverages feature maps from the final convolution layer to capture spatial details essential for identifying visual patterns and class assignments, offering insights into what parts of an image most affected the model’s labeling.

### 2.5. Benchmark Model, Model Testing, and Statistical Methods

The Rapid Shallow Breathing Index (RSBI) was chosen as the benchmark to compare model performance given its long history and widespread use as a weaning predictor [5]. The last RSBI value before the extubation time was kept for each patient, with a widely used cutoff value of RSBI under 105 breaths/min/L predicting extubation success. To generate the Receiver Operating Curve for the RSBI score, probability scores were generated after scaling the RSBI values using MinMax Scalar method in Scikit-learn in Python.

For each of the developed transfer learning models, performance was evaluated on the test set and on the holdout set (which was not used for model development), and the model-derived class probabilities were used to predict extubation success within 72 h. The prediction threshold is selected such that there is a balance between sensitivity and specificity. Predictions less than the threshold were categorized as negative. Sensitivity, specificity, accuracy, positive predictive value (PPV), negative predictive value (NPV), F1 score, AUROC, and area under the precision–recall curve (AUPRC), along with bootstrap 95% CIs, were estimated for evaluating the screening tool’s performance. For demographics, group comparisons were performed using Student’s t-test or Kruskal–Wallis for continuous variables as appropriate, and the chi-squared test for categorical variables. All analysis was performed using SciPy [17].

## 3. Results

### 3.1. Study Population and Outcomes

A total of 2288 intubated patients were included in the overall study cohort; clinical characteristics and demographics are summarized in Table 1. In total, 52% of the overall cohort was male, and the median age was 63.3 years. The median duration of ICU length of stay was 4.7 days and ranged between 0.1 and 37.8 days. The overall rate of extubation success was 88.2% in the whole study cohort. There was no statistically significant difference between patients with extubation success and patients with extubation failure for all key characteristics, except for the ICU length of stay: patients with an extubation failure stayed significantly longer than patients with an extubation success (*p* < 0.001).

### 3.2. Final Imaging Classifier and Predictors in the Imaging Classifier

**Figure 2:**
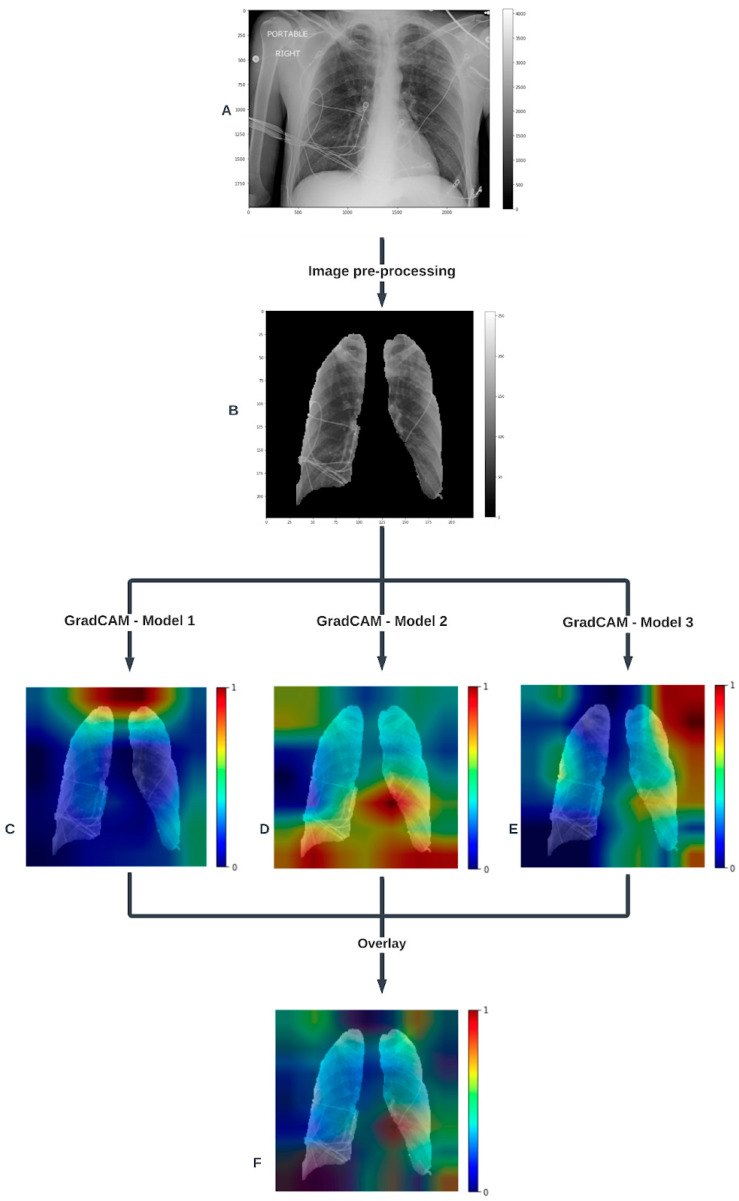
Flow chart of the imaging pipeline with one example chest X-ray. It starts from the raw chest X-ray (**A**). (**B**) shows the segmented and processed chest X-ray resulting from the processing pipeline. The most important features (pixels) predicting extubation success are highlighted in the class activation map calculated by Grad-CAM projected on the image for the top 3 models (**C**–**E**). Lastly, (**F**) shows the superimposed Grad-CAM image in the final model.

The final model was an ensemble of the three highest-performing cross-validation models with final probability scores averaged to produce a final prediction (Figure 2). The Grad-CAM (Gradient-weighted Class Activation Mapping) technique enables us to visualize which specific regions of an image significantly influenced the model’s decision for a given label. By utilizing the gradients of the target label (extubation success) that flow into the final convolutional layer, Grad-CAM generates a coarse localization map that highlights important areas within the image for label prediction. As illustrated in Figure 2, for various individual models, the regions that most strongly contributed to predicting the target label are indicated by the red-highlighted areas on the segmented images. These red regions represent the critical parts of the image where the model focused its attention when making the prediction. When the Grad-CAM visualizations from different models are superimposed, the composite image shows the predominant areas that influenced the final average model’s decision, highlighted in red.

Because the final probability prediction is an average of three distinct models’ probability predictions, the Grad-CAM visualizations from each independent model were first generated, and then all Grad-CAM images from all models were superimposed in the final visualization. Figure 2 shows the image processing flow chart and individual models’ Grad-CAM images as well as the final superimposed Grad-CAM image. 

### 3.3. Predictive Performance of the Model

At a prediction probability threshold of 0.81, the final image classifier provided a sensitivity of 58% (95% CI: 55–62%), specificity of 87% (95% CI: 84–89%), accuracy of 73% (95% CI: 71–75%), and AUC-ROC of 0.87 (95% CI: 0.85–0.89) on the training set. On the testing set, it reached a sensitivity of 62% (95% CI: 56–69%), specificity of 60% (95% CI: 39–79%), accuracy of 62% (95% CI: 60–68%), and AUC-ROC of 0.66 (95% CI: 0.54–0.76).

**Figure 3:**
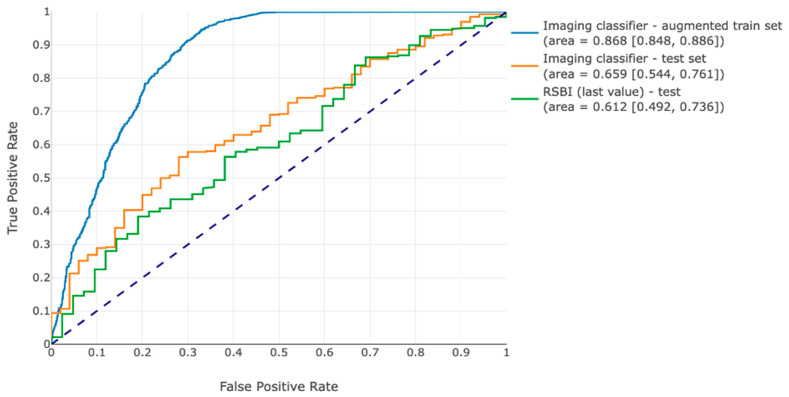
Receiver operating curves for the image classifier on the training set and testing set, as well as the RSBI in the test set.

**Figure A1:**
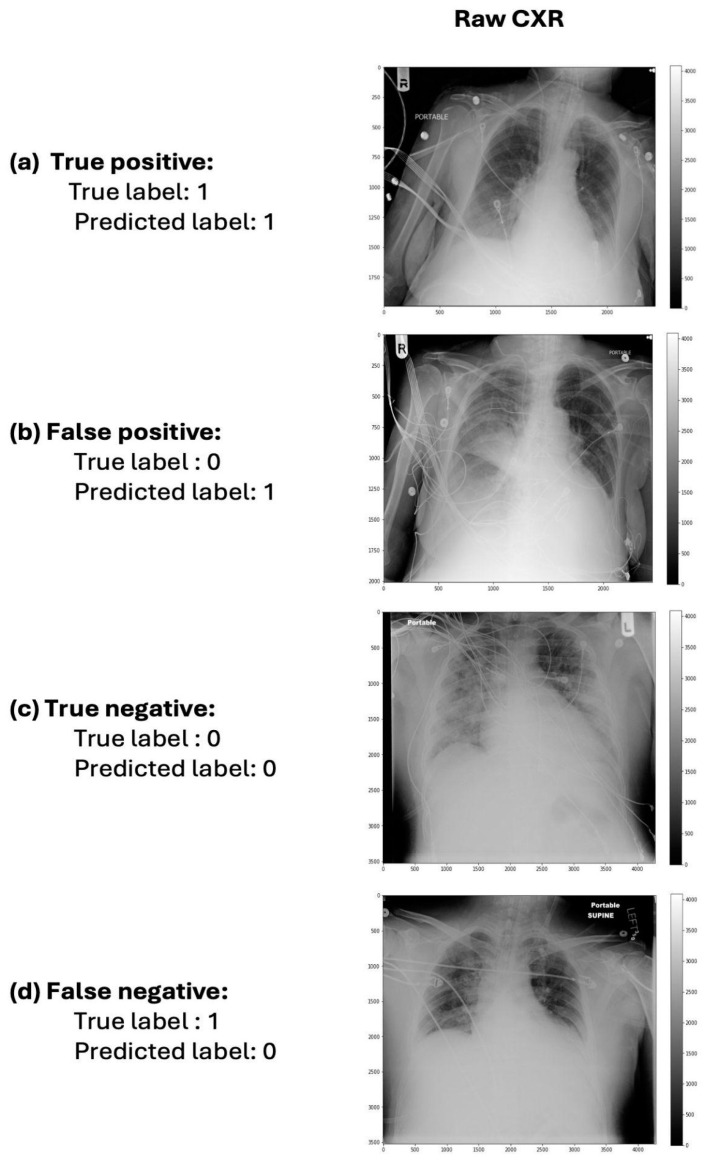
Examples of input images and classification outcomes demonstrating (**a**) a true positive successful extubation, (**b**) a false positive prediction, (**c**) a true negative prediction, and (**d**) a false negative prediction. A label of 1 signifies successful extubation at 72 h; a label of 0 signifies extubation failure by 72 h.

Compared to the benchmark RSBI score, the image classifier gave boosted performance results in the test set in terms of specificity (from 16% to 60%), PPV (from 90% to 93%), and AUROC (from 0.61 to 0.66), and a similar AUPR (0.93 vs. 0.94). Table 2 shows all performance metrics for the imaging model and the RSBI benchmark on the test set. Receiver operating characteristic (ROC) curves are shown in Figure 3. Example images for true positive, false positive, true negative, and false negative predictions are presented in Appendix A, Figure A1. 

## 4. Discussion

In the current study, we develop and internally validate a deep learning model using routinely available chest radiographs for the prediction of extubation outcomes in medically critically ill patients undergoing invasive mechanical ventilation. The optimized model was an ensemble of the highest-performing trained ResNet models, in which probability scores were averaged amongst the three, resulting in a more balanced probability distribution and more even sensitivity and specificity than from any single model alone. The final model achieved an AUC of 0.66 and an AUPRC of 0.94, as well as a sensitivity and specificity of 0.62 and 0.60, respectively, when calibrated to a prediction threshold of 0.81. In our testing dataset, the model performs better than the RSBI with higher specificity, precision, and AUC-ROC. The final classifier has improved performance compared to other work in this space (AUC 0.55) [11]. In the current model, there remains a large gap between training performance (AUC 0.87) and testing performance (AUC 0.67), suggesting overfitting and potential for even further model improvements with more training examples and the use of a pre-training dataset more closely aligned to CXRs, as opposed to ImageNet.

CXRs have been consistently regarded as key to identifying improvement in the cause of respiratory failure, driving the need for mechanical ventilation. Because of their nature as an image and not an easily quantifiable numeric score, there is limited research on the use of chest radiographs in extubation prediction [1]. One early attempt to incorporate chest X-rays into a systematic weaning program was the Burns Modified Weaning Program, which simply asks whether the patient’s X-ray is improving or not, but this does not incorporate the breadth of information contained in the image [18].

In the age of deep learning, we can now overcome these barriers and incorporate chest X-rays into multimodal predictions. There is a robust base of evidence that chest X-rays may be useful in predicting the need for intubation in patients not yet on mechanical ventilation, especially during the COVID-19 pandemic, with trends for higher performance among larger datasets and those models pre-trained on ChexNeXt as opposed to ImageNet [19,20,21,22,23,24]. On the other hand, the use of chest X-rays in extubation prediction is an open problem.

To our knowledge, there is only one other study describing the use of chest radiographs in weaning prediction from Fukuchi et al., in which they use n = 1066 episodes of invasive mechanical ventilation in the MIMIC-IV dataset with a similar study design to the current work. In their study, they found that the deep learning model trained on CXRs was unable to perform better than the RSBI, and the CXR classifier alone had an AUC of 0.55 (95% CI 0.49–0.6) with a sensitivity of 66% and specificity of 44%. These suggest performance similar to a no-skill classifier, and they concluded that the use of CXRs did not improve the prediction of extubation outcome. There are several major differences between their study and the present study, including defining extubation success at 48 h as opposed to 72 h here and using EfficientNet as a model architecture; however, the two factors that seem to contribute the most are a significantly higher sample size (*n* = 2288 vs. *n* = 1066) and the use of segmented images with meticulous pre-processing in the current study. Steps taken in the current pipeline, including histogram matching to regularize exposure, isolating the key ROI with a segmentation algorithm, and centroid alignment, ensure that only the most useful information is retained, noise is limited, and precise and reliable images are passed to the image classifier. Fukuchi’s group does not report the cross-validation training performance or the size of their train/test split; however, the results of the current study suggest that increasing training size may optimize model performance.

Grad-CAM was used to identify regions of interest influencing model output to provide a degree of explainability to model predictions. Because earlier work in this space did not employ this technique, and extubation success or failure is a complex phenotype and not a specific imaging finding, it is not known which regions are expected to drive predictions in this population. Figure 2 demonstrates the Grad-CAM images of the top three models and the final ensemble model; conserved regions of interest among the three models are at the left hilum and lung apices. While there is no specific literature to suggest findings in these areas are more or less likely to influence ventilator liberation, it is interesting to note that a similar distribution was found in a CXR transfer learning study using routinely available CXRs to classify patients with Chronic Obstructive Pulmonary Disease (COPD) [25]. While this is a different label than the current study, it is worth noting that it too is a complex phenotype as opposed to a direct imaging finding, and it is possible that the deep learning models are picking up changes in structures like pulmonary vasculature or retrocardiac opacities that human reviewers may not identify. Finally, some of the regions on the Grad-CAM map have no expected contribution to the prediction, including the image corners where no segmented lung is present. These are likely remnants from the pre-training of the model on the ImageNet dataset, in which these areas may have been more important. A larger transfer learning cohort or training the model from scratch on lung images would be expected to address this issue.

The present study has several strengths, including the development and deployment of an end-to-end automated pipeline for X-ray pre-processing and model prediction, which allows for a seamless transition to prospective evaluation; the use of segmented images; the use of real-world clinical labels for extubation success or failure; the use of Grad-CAM to help identify regions of interest in the final output; and higher performance compared to current benchmarks and previous work. At the same time, the current work has several limitations and much further exploration is warranted. The current model was trained at a single center in a single ICU, and it remains to be seen how well it will generalize to future or different patient populations. Furthermore, the model was trained with a very small dataset, resulting in overfitting with a drop in performance from training to testing. The model was pre-trained on ImageNet for convenience; however, pre-training a model on a medical imaging dataset would potentially improve performance in this domain. These factors contribute to the relative drop in performance from training to testing; these represent next steps for improvement in model development in this space. Finally, it remains to be seen how the model will interact with EMR features, and the best method to combine these two different modalities (images and tabular data) remains unknown [26]. Future work will have to address these limitations.

## 5. Conclusions

As the availability of deep learning and the integration of machine learning pipelines in healthcare improve, the use of multimodal data in clinical decision support is the next frontier. Here, we demonstrate that CXR images using deep learning can predict the outcome of a trial of extubation with performance similar to or higher than existing benchmarks or previous work, though there remains significant room for improvement and optimization. The left hilum and lung apices were identified as regions of interest in the final model; however, the significance of these findings requires further investigation. Future work will involve improving model pre-training, increasing the size of the training cohort, and optimizing performance in the hope of one day reaching clinical deployment.

## Figures and Tables

**Table 1:** Clinical characteristics and demographics of the study cohort.

	Overall *(n =* 2288)	Extubation Success *(n* = 2017, 88.2%)	Extubation Failure *(n* = 271, 11.8%)	*p*-Value
**Age**				0.77
Mean (SD)	61.8 (16.4)	61.9 (16.4)	61.6 (16.2)	
Median [Min, Max]	63.3 [18, 104]	63.4 [18, 104]	51.8 [20, 101]	
**Gender**				0.52
Male	1195 (52.2%)	1048 (52.0%)	147 (54.2%)	
Female	1093 (47.8%)	969 (48.0%)	124 (45.8%)	
**Race and Ethnicity**				0.15
White	704 (30.8%)	623 (30.9%)	81 (29.9%)	
African American	561 (24.5%)	495 (24.5%)	66 (24.4%)	
Hispanic	250 (10.9%)	231 (11.5%)	19 (7.0%)	
Asian	99 (4.3%)	89 (4.4%)	10 (3.7%)	
Other	514 (22.5%)	440 (21.8%)	74 (27.3%)	
Unspecified	160 (7.0%)	139 (6.9%)	21 (7.7%)	
**BMI**				0.23
Mean (SD)	27.9 (9.8)	27.8 (9.7)	28.7 (10.9)	
Median [Min, Max]	28.3 [10.8, 181.8]	25.9 [10.8, 181.8]	26.8 [14.3, 130.6]	
**Ideal Body Weight**				0.87
Mean (SD)	60.7 (11.1)	60.8 (11.2)	60.6 [10.6]	
Median [Min, Max]	59.6 [36.0, 98.3]	59.3 [36, 98.3]	61.5 [36.0, 85.0]	
**Smoking history**				0.53
Current Smoker	50 (2.2%)	44 (2.2%)	6 (2.2%)	
Past smoker	675 (29.5%)	603 (29.9%)	72 (26.6%)	
Never smoked	252 (11.0%)	216 (10.7%)	36 (13.3%)	
Missing	1311 (57.3%)	1154 (57.2%)	157 (57.9%)	
**Hypertension**				0.32
Yes	754 (33.0%)	670 (33.2%)	84 (31.0%)	
No	407 (17.8%)	350 (17.4%)	57 (21.0%)	
Missing	1127 (49.3%)	997 (49.4%)	130 (48.0%)	
**Diabetes**				0.9
Yes	447 (19.5%)	393 (19.5%)	54 (20.0%)	
No	714 (31.2%)	627 (31.1%)	87 (32.0%)	
Missing	1127 (49.3%)	997 (49.4%)	130 (48.0%)	
**COPD**				0.71
Yes	366 (16.0%)	318 (15.8%)	48 (17.7%)	
No	795 (34.7%)	702 (34.8%)	93 (34.3%)	
Missing	1127 (49.3%)	997 (49.4%)	130 (48.0%)	
**Obesity**				0.29
Yes	226 (9.8%)	192 (9.5%)	34 (12.5%)	
No	935 (40.9%)	828 (41.1%)	107 (34.5%)	
Missing	1127 (49.3%)	997 (49.4%)	130 (48.0%)	
**ICU Length of Stay (days)**				<0.001
Mean (SD)	6.2 (5.0)	5.5 (4.4)	11.1 (6.2)	
Median [Min, Max]	4.7 [0.1–37.8]	4.3 [0.1–37.7]	10.3 [0.1–37.8]	

**Table 2:** Summary performance of the image classifier and the Rapid Shallow Breathing Index on the test set.

Model Name	Sensitivity	Specificity	Accuracy	PPV	NPV	F1 Score	AUROC	AUPRC
RSBI Benchmark	0.92 [0.88, 0.96]	0.16 [0.04, 0.33]	0.84 [0.79, 0.88]	0.90 [0.85, 0.94]	0.20 [0.05, 0.39]	0.91 [0.88, 0.94]	0.61 [0.49, 0.73]	0.93 [0.87, 0.96]
Imaging classifier	0.62 [0.56, 0.69]	0.60 [0.39, 0.79]	0.62 [0.60, 0.68]	0.93 [0.88, 0.97]	0.17 [0.09, 0.25]	0.75 [0.69, 0.80]	0.66 [0.54, 0.76]	0.94 [0.90, 0.97]

## Data Availability

The raw data underlying this article were generated at the Mount Sinai Health System. Derived data supporting the findings may be available from the corresponding author (P.T. (Pranai Tandon)) upon request.

## Appendix A

Representative DICOM images for true positive, false positive, true negative, and false negative images are provided in Figure A1. While certain findings are apparent in the images, such as right middle lobe collapse in a false positive (Figure A1b) or airspace opacities in a true negative (Figure A1c), caution must be taken in their interpretation because the prediction of extubation success or failure at least 72 h after the image was captured is a more complex phenotype than directly identifying diagnostic abnormalities on the image itself. Because all patients included in this study were extubated by ICU clinicians who reviewed the images, features driving classification may not be readily apparent on clinical review. Instead, the model may incorporate more subtle features and different kinds of information than what is readily visible to the human eye. More discussion regarding the use of Grad-CAM to identify regions of interest driving the model predictions is presented in the main text.
